# Timing of erythropoietin modified mesenchymal stromal cell transplantation for the treatment of experimental bronchopulmonary dysplasia

**DOI:** 10.1111/jcmm.13843

**Published:** 2018-08-30

**Authors:** Zhaohua Zhang, Chao Sun, Jue Wang, Wen Jiang, Qian Xin, Yun Luan

**Affiliations:** ^1^ Department of Pediatrics The Second Hospital of Shandong University Jinan China; ^2^ Central Research Laboratory Institute of Medical Science The Second Hospital of Shandong University Jinan China

**Keywords:** bronchopulmonary dysplasia, erythropoietin gene modified mesenchymal stem cells, Inflammation, p38 MAPK

## Abstract

The aim of this study is to optimize the timing of erythropoietin gene modified mesenchymal stem cells (EPO‐MSCs) transplantation for bronchopulmonary dysplasia (BPD). Three weeks post‐operation, the results indicated that the damage of airway structure and apoptosis were significantly decreased, the proliferation was increased in three EPO‐MSCs transplantation groups as compared with BPD mice. Moreover, the inflammation cytokines were improvement in early EPO‐MSCs injection mice than in BPD mice, but there was no significant difference between late injection and BPD groups. Furthermore, the protein expression ratio of p‐p38/p38MAPK was down‐regulation in early mice but not in late transplantation mice. Our findings suggest that EPO‐MSCs maybe attenuate BPD injury in early than in late administration by inhibiting inflammation response through down‐regulation of the p38MAPK signalling pathway.

## 1 INTRODUCTION

Mesenchymal stromal/stem cells (MSCs) therapy has been shown in mouse and rat models of bronchopulmonary dysplasia (BPD), the protection mechanism is through anti‐inflammation and improvement alveolar structure rather than regeneration.^1^ However, the narrow therapeutic time window limits its application. In the previous study, we showed that bone‐derived MSCs in combination with erythropoietin (EPO) can significantly reduce the newborn mice alveolar injury and lung fibrosis induced by high oxygen than MSCs therapy alone,^2^ but the transplant timing has not been reported. In this study, we will investigate the EPO gene‐modified MSCs transplant time‐point for treatment of BPD.

## 2 MATERIALS AND METHODS

### 2.1 Cell culture and EPO gene‐modified MSCs

Mesenchymal stromal/stem cells were isolated from the tibia and femurs of all four limbs of C57BL/6 mice using whole bone marrow culture method. EPO gene modified BMSCs were established as previously reported.^3^

### 2.2 Animal model and cells transplantation

All animal procedures were approved by the animal ethics committee of Shandong University (Jinan, China). BPD model was established as previously described with some modifications.^4^ EPO‐MSCs (1 × 10^6^) were injected intravenously at 1‐day (D1) and 7‐day (D7) after exposure to high oxygen. Animals were divided into 5 groups: normal group, hyperoxia group (BPD), hyperoxia with early (D1), late (D7) and early+late combined transplantation (D1 + 7) group.

### 2.3 Histology

Three weeks after operation, animals were anaesthetized and the left upper lobe was removed. Tissue samples were embedded in paraffin and were stained with haematoxylin and eosin (H&E). Terminal dUTP nick end‐labelling (TUNEL) assay and Ki67 staining were performed to measure the apoptosis and proliferation. Fluorescent images were taken with a Nikon Eclipse 90i microscope (Nikon Corporation, Tokyo, Japan) and the merged picture using the image‐analysis system Image‐Pro Plus 6.0 (Media Cybernetics, Rockville, MD, USA).

### 2.4 Quantitative real‐time PCR

The right lung was frozen in liquid nitrogen and stored at −80°C. Total RNA was extracted from frozen samples, qRT‐PCR analysis was performed to detect the relative mRNA levels of TNF‐a, IL‐1, IL‐6, IL‐8 and IL‐10 in the lung. Relative quantification of gene expression was performed using the comparative threshold cycle (ΔΔCt) method.

### 2.5 Western blot analysis

The frozen right lung tissue was lysed using protein extraction buffer and the protein expression of p38MAPK and p‐p38MAPK were detected by Western blot. Specific primary antibodies p38MAPK and p‐p38MAPK monoclonal antibody were used, and goat anti‐rabbit IgG (Boshide Inc., Shanghai, China) were incubated as the secondary antibody.

## 3 RESULT

### 3.1 Characterization of cultured MSCs

After being primarily cultured for 3 days, the MSCs appeared as spindle‐like cells and attached to the tissue culture dishes. Three days after being subcultured, the cells were attached to the culture dish tightly and proliferated rapidly in the culture medium. The surface markers of MSCs were determined by FACS, The cells indicated expression of the surface markers CD44 (95.6%), CD90 (98.3%) and CD106 (96.7%) negative expression of the haematopoietic markers CD45 (0.5%) and CD34 (0.8%) and CD117 (0.1%). On the other hand, in vitro differentiation capacity of MSCs was examined to investigate further the potency of MSCs. The results showed that the cells have the ability of adipogenic and osteogenic.

### 3.2 Bodyweight gain

**Figure 1:**
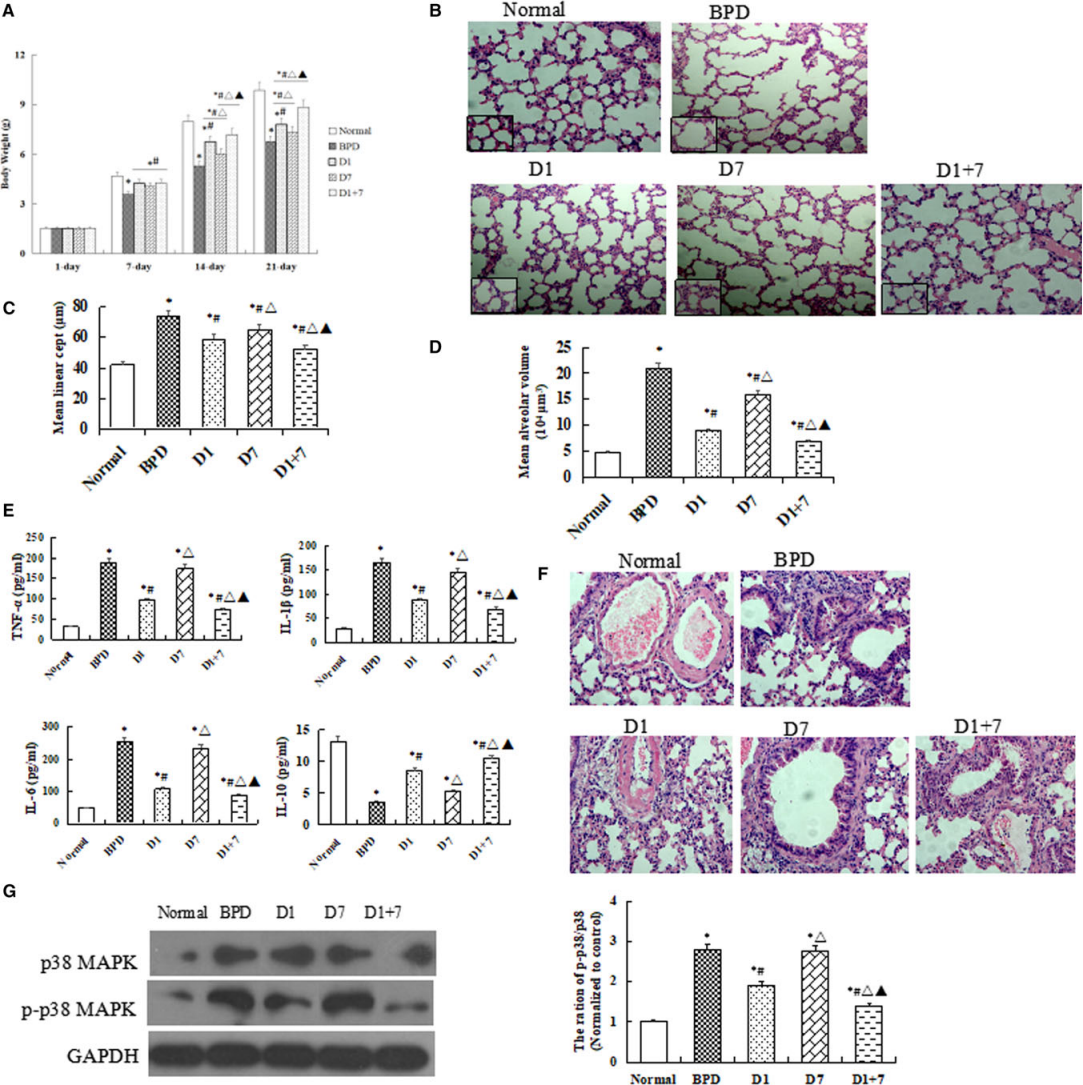
Body weight, tissue injury and inflammation response detection. A, The average bodyweight (g). B, HE staining. C, alveolarization measured by the mean linear intercept and D, mean alveolar volume. E, mRNA levels of TNF‐α, IL‐1β, IL‐6 and IL‐10. F, Inflammation response around the pulmonary vascular. G, the protein expression of p38MAPK and p‐p38MAPK.The data are present as mean±SD. *P <* *0.05 vs Normal; ^#^ *P *<* *0.05 vs BPD group. ^▵^ *P *<* *0.05 vs D1 group. ^▲^ *P *<* *0.05 vs D7 group

As shown in Figure 1A, the bodyweight was increased in all three EPO‐MSCs injection groups than that in BPD group (*P *<* *0.05). Importantly, the increased is more significantly in both D1 and D1 + 7 groups when compared with D7 group, especially in D1 + 7 group (*P *<* *0.05).

### 3.3 Lung histopathology

H&E staining showed that EPO‐MSCs injection groups have smaller and more numerous alveoli than BPD group in lung tissue. The mean linear intercept and mean alveolar volume were decreased in treatment groups than in BPD group, particularly, there was a significantly lower in D1 group as compared with D7 group (*P *<* *0.05, Figure 1B‐D).

### 3.4 Inflammatory responses

qRT‐PCR results showed that mRNA levels of TNF‐α, IL‐1β and IL‐6 were lower, but IL‐10 was higher in EPO‐MSCs injection groups than in BPD group, a more obvious change was shown in D1 + 7 group (*P *<* *0.05, Figure 1E). As shown in Figure 1F, a significant correlation can be drawn between the intensity of the inflammation around the pulmonary vascular. Moreover, our Western blot results showed that the protein expression of p38MAPK and p‐p38 MAPK in lung tissue were significantly lower in D1 and D1 + 7, mice than in BPD mice, but there was no difference between D7 and BPD group **(**Figure 1G).

### 3.5 Cell apoptosis and proliferation

**Figure 2:**
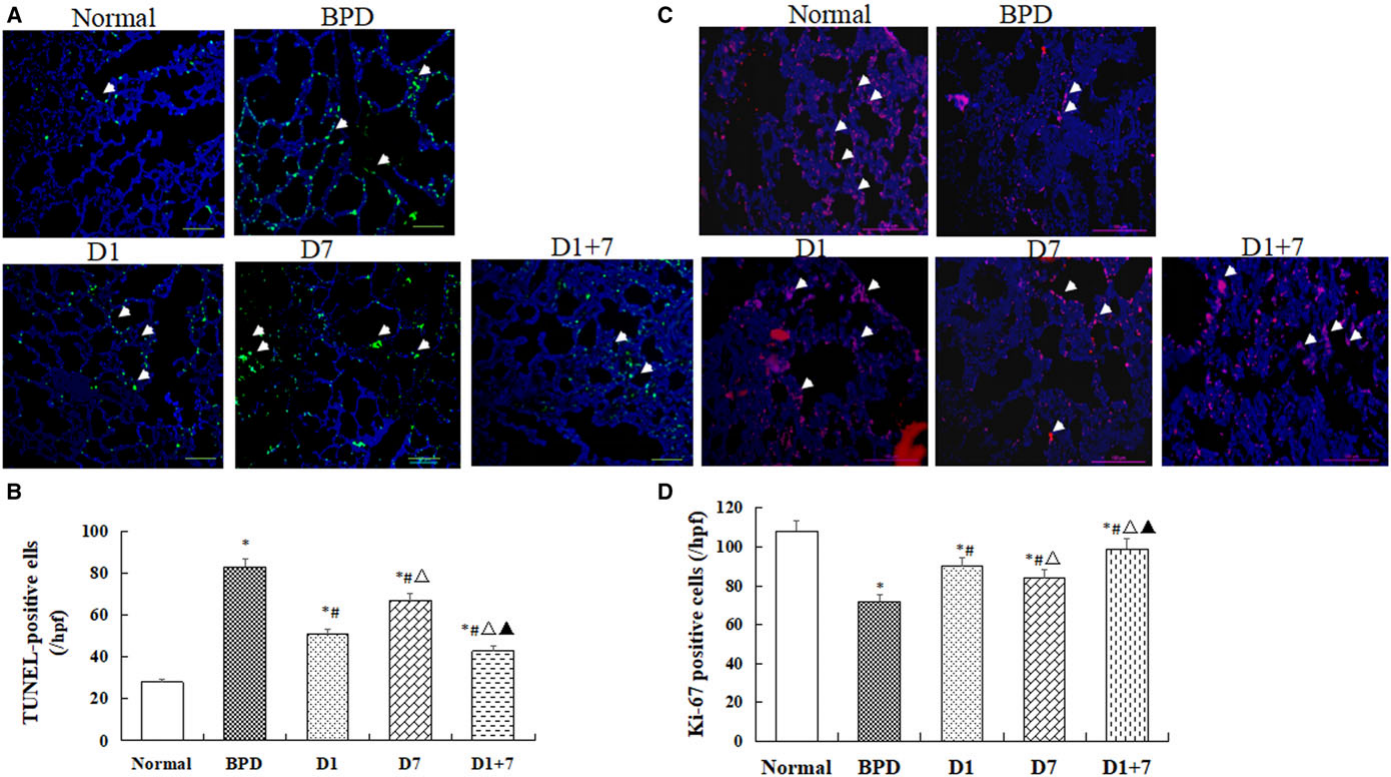
Cell apoptosis and proliferation analysis. A, TUNEL‐positive cells were labelled with FITC (green) and the cell nuclei were labelled with DAPI (blue) using a light microscope at a ×100 magnification. B, A comparative analysis of number of TUNEL‐positive cells in each group. C, Cells proliferation detected by Ki67 staining (red) and the cell nuclei were labelled with DAPI (blue) using a light microscope at a × 200. D, A comparative analysis of number of Ki‐67 positive cells in each group. The data are present as mean±SD. *P <* *0.05 vs Normal; ^#^ *P *<* *0.05 vs BPD group. ^▵^ *P *<* *0.05 vs D1 group. ^▲^ *P *<* *0.05 vs D7 group

As shown in (Figure 2A,B), the number of TUNEL‐positive cells was increased in lung tissue in BPD mice than in the normal mice, but it was decreased in three EPO‐MSCs treatment groups. There was a significantly lower in D1 especially in D1 + 7 groups than D7 group (*P *<* *0.05). On the contrary, the count of Ki‐67‐positive cells was increased in three EPO‐MSCs treatment groups, especially in D1 and D1 + 7 groups than in BPD group (*P *<* *0.05, Figure 2C,D).

## 4 DISCUSSION

Stem cell transplantation has become a potential new treatment methods in animals models of BPD and in phase 1 or early‐phase clinical trials; it can migrate to lung tissue.^5^, ^6^, ^7^ Reports showed that EPO can stimulate differentiation and proliferation of erythroid progenitor cells.^8^ The present study, shows that the therapeutic efficacy of EPO gene‐modified MSCs for BPD is time‐dependent, the early transplantation is better than the late.

TGF‐β1 signalling pathway plays an important role in lung development,^9^ and TGF‐β‐positive myofibroblasts levels are increased in BPD.^10^ Overexpression of TGF‐β1 lead to abnormal alveolar structure, and vascular development in neonatal mouse lungs.^11^ We have shown that MSCs in combination with EPO could attenuate lung fibrosis induced by high oxygen through inhibition of TGF‐β1/Smad signalling.^2^ Recent studies suggest that inflammation response also plays a major role in the development of BPD,^12^ therefore, it is very necessary to study whether the MSCs and EPO combination therapy can more attenuate inflammation than MSCs transplantation alone. p38 MAPK acts upstream of LPS‐induced nuclear factor‐kappa B (NF‐κB), which is a key participant in the inflammation, immune response, and regulation of cell differentiation and apoptosis during acute lung injury.^13^, ^14^, ^15^ However, the report about p38MAPK in high oxygen‐induced lung injury is very rare. Interestingly, our present results indicated that the protein expression of p38MAPK and p‐p38MAPK were significantly lower in early transplantation group, but not in late transplantation group.

Reports have found that MSCs transplantation in the treatment of BPD mainly through paracrine role, more recently, using experimental models of BPD have shown that exosomes are the responsible therapeutic vector for the “main” therapeutic effects afforded by MSCs.^4^ On the other hand, considering the saccular stage of lung development and the lung development process, choose postnatal day 4 as a treatment time seems to be very necessary. Those cannot be clarified in the present study and would need further investigations.

Collectively, we report the proof of principle demonstration that EPO‐MSCs administration can significantly attenuate BPD neonatal mice lung injury in early than in late transplantation, the underlying mechanism maybe through inhibition of inflammation by down‐regulation of the p38MAPK signalling pathway, and further studies would be useful.

## CONFLICT OF INTEREST

The authors confirm that there are no conflict of interests.
